# Molecular Insights Into the Role of Gut Microbiota in Antibiotic Therapy Selection and Resistance Mitigation

**DOI:** 10.7759/cureus.50318

**Published:** 2023-12-11

**Authors:** Mihaela Andreescu

**Affiliations:** 1 Hematology, Colentina Clinical Hospital, Bucharest, ROU

**Keywords:** prognosis, early detection, clinical diagnostic, infectious diseases, molecular biology

## Abstract

Antibiotic therapy is a cornerstone of modern medicine, yet the development of antibiotic resistance threatens to render these therapies ineffective. The gut microbiota, a complex ecosystem of microorganisms residing in the gastrointestinal tract, plays a critical role in modulating antibiotic efficacy and resistance. This review delves into the intricate relationship between gut microbiota, antibiotic therapy, and resistance and discusses the potential applications of gut microbiota research in guiding personalized antibiotic therapy and resistance mitigation strategies. Recent advancements in metagenomics, metatranscriptomics, and metabolomics have demonstrated the potential for tailored antibiotic regimens that minimize collateral damage to commensal bacteria and reduce the risk of resistance. Adjuvant therapies, such as probiotics, prebiotics, and synbiotics, have shown promise in restoring gut microbial balance and mitigating the adverse effects of antibiotic therapy. We address the challenges associated with this emerging field, including the need for standardized methodologies, ethical considerations, and interdisciplinary collaboration. With continued interdisciplinary collaboration and the implementation of standardized methodologies, gut microbiota research can contribute to the global fight against antibiotic resistance and improve patient outcomes.

## Introduction and background

Antibiotic resistance has emerged as a significant concern for public health, which is likely to jeopardize the efficacy of life-saving antimicrobial treatments [1,2]. The inappropriate use of antibiotic results in the development of antimicrobial resistance. Multidrug-resistant organisms are increasingly recognized as a significant source of healthcare-associated infections. The World Health Organization (WHO) has identified antibiotic resistance as one of the most pressing global health threats, with an estimated 700,000 deaths occurring annually due to drug-resistant infections. Failure to address this problem is expected to lead to a projected increase in the annual death toll to 10 million by 2050 [3]. Furthermore, the misuse or overuse of some antibiotics have exacerbated the situation [4]. We are also seeing a decline in investments in antibiotic research, which is visible in the form of slow development of new antibiotics [5,6].

Amidst these concerns, the gut microbiota has gained significant attention as a crucial modulator of host health and disease [7]. The human gut microbiota has a unique significance in human biology as it comprises trillions of microorganisms, including bacteria, viruses, and fungi. Recent research has demonstrated that the gut microbiota exerts a profound influence on various aspects of human physiology, including metabolism, immunity, and neurological function [8]. The role of gut microbiota and brain has been extensively studied due to its significant role in human emotions and psychological health [9,10]. In 2013, Clarke et al. reported that early-life microbiota regulates the hippocampal serotonergic system, which opened up multiple line of inquiries into the role of microbiome on neurodevelopment [11].

The intricate interplay between the gut microbiota and the host immune system is essential to maintain homeostasis for the optimal functioning of an individual. Imbalances in this interplay have been linked to a range of diseases, including infectious diseases [12]. Therefore, understanding the intricate relationship between the gut microbiota, antibiotic therapy selection, and resistance mitigation is of paramount importance for disease management. Thanks to the advancements in molecular biology techniques, such as metagenomics, transcriptomics, and metabolomics, the composition of function of gut microbiota is becoming more clearer with each passing day [13]. These cutting-edge technologies have shed light on the intricate interplay between the gut microbiota and the pharmacokinetics and pharmacodynamics of antibiotics [14].

Furthermore, newer research has highlighted the potential of the gut microbiota to overcome antibiotic resistance through competitive exclusion, colonization resistance, and modulation of host immune responses [15-17]. The emergence of next-generation sequencing (NGS) technologies has transformed the field of microbiome research, which has enabled comprehensive and high-resolution characterization of the gut microbial community [18]. Several studies have explored the potential of manipulating the gut microbiota to optimize antibiotic therapy and mitigate resistance [19-21]. The use of prebiotics, probiotics, and fecal microbiota transplantation (FMT) are some examples of interventions that have shown promise in modulating the gut microbiota, with potential implications for antibiotic therapy selection and resistance mitigation [22,23]. Moreover, the integration of gut microbiota research into clinical decision-making is beginning to gain traction, as evidenced by the increasing number of clinical trials and studies investigating the use of gut microbiota information to guide antibiotic therapy [24-26].

The present review presents an extensive overview of the molecular mechanisms underlying the influence of gut microbiota on the selection of antibiotic therapy and the alleviation of antibiotic resistance. The article emphasizes the clinical significance, hurdles, and potential research pathways concerning gut microbiota in the context of infectious diseases. The knowledge in this review will improve clinical decision-making and help design innovative therapeutic strategies.

## Review

Gut microbiota and antibiotic therapy selection

The gut microbiota can significantly influence the efficacy and toxicity of orally administered antibiotics as it plays a crucial role in modulation of their pharmacokinetics and pharmacodynamics. Pharmacokinetics refers to the absorption, distribution, metabolism, and excretion of drugs, while pharmacodynamics deals with the relationship between drug concentration and its effect on the body [27]. Both aspects are critical in determining the success of antibiotic therapy.

**Figure 1:**
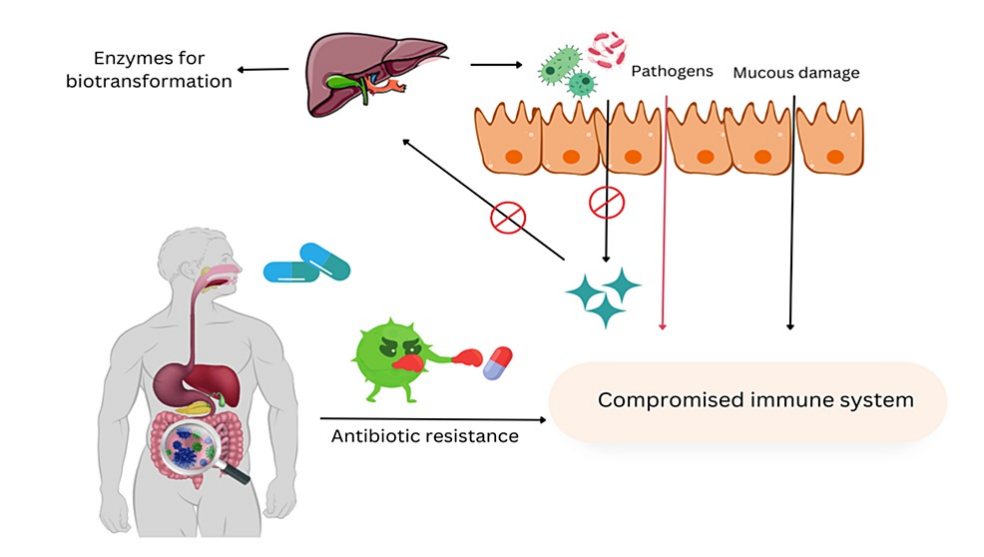
Negative effects of antibiotic misuse on the host health Figure 1 shows the sequence of events that takes place in the case of antibiotic misuse. In the first step, antibiotic resistance develops that leads to a compromised immune system. A major impact is seen on a protective mucin that is damaged. Consequently, there is an influx of pathogens and blockage of healthy bacteria.

The gut microbiota also influences the absorption of antibiotics by metabolizing the drug or altering the intestinal environment, which can lead to changes in drug bioavailability [28]. For instance, certain bacterial species in the gut can metabolize beta-lactam antibiotics, which can render them inactive and reduce their efficacy. Furthermore, the gut microbiota can affect drug distribution by altering the expression of efflux transporters and drug-metabolizing enzymes in the intestinal epithelium [29]. These alterations can significantly affect the systemic availability and tissue distribution of antibiotics, which can ultimately impact their efficacy and toxicity [30]. The gut microbiota can also influence the bile acid pool, which can affect the solubility and absorption of antibiotics [31]. The gut microbiota can also impact the pharmacodynamics of antibiotics by modulating the host immune response (Figure 1). Several studies have revealed that the gut microbiota plays a crucial role in regulating both the innate and adaptive immune responses, which are critical to control and resolve bacterial infections [32]. The gut microbiota can facilitate the efficacy of specific antibiotics through the promotion of antimicrobial peptide production and regulation of immune cell differentiation and activation [33]. Furthermore, the gut microbiota is capable of generating short-chain fatty acids that enhance immune cell function and amplify the effectiveness of antibiotics against bacterial infections [34]. However, it is important to note that certain gut bacteria can produce enzymes that disable antibiotics, which can reduce their potency and promote the development of antibiotic resistance [35].

One of the key examples illustrating the impact of the gut microbiota on antibiotic therapy selection is the interaction between the gut microbiota and the antibiotic vancomycin. The gut microbiota can modulate the pharmacokinetics and pharmacodynamics of vancomycin by altering its absorption, distribution, and metabolism, as well as its ability to elicit an immune response [36]. Furthermore, gut microbiota dysbiosis caused by vancomycin treatment has been associated with increased susceptibility to *Clostridiodes difficile* infection. A study by Buffie et al. reported that the administration of a single clindamycin dose resulted in a significant reduction in the diversity of the intestinal microbiota for a minimum of 28 days. Moreover, it caused a long-term reduction of approximately 90% of the normal microbial taxa present in the cecum [37].

An example of persisters is *Staphylococcus aureus*, which acquires antibiotic resistance easily. Even the most susceptible *S. aureus* survives and persists the antibiotic therapy. It requires prolonged surgical interventions and treatments. They tolerate high concentrations of antibiotics, display the phenotype of arrested growth, and are are linked with recurrent and chronic infections [38]. Huemer et al. in their study characterized these persisters. Multiomics analysis recognized molecular fluctuations in *S. aureus* in reaction to acid stress, paving to a general infectious population. They persist also due to molecular reprogramming, such as ribosomal protein upregulation, downregulation of virulence, amino acid pathways, cell division, ATP levels, lowered aconitase activity, and accretion of insoluble proteins used in translation, transcription, and energy production. They showed that a directed antipersister therapy by the use of retinoid byproducts and antibiotics helps to eliminate persistiors to some extent [39].

Furthermore, it has been shown that the gut microbiota can contribute to antibiotic-related toxicity. Antibiotics can disrupt the gut microbiota, which leads to the overgrowth of pathogenic bacteria, such as *Clostridium difficile*, an etiological factor for severe diarrhea and colitis [33]. In addition, alterations in the gut microbiota can lead to changes in the production of microbial metabolites, such as indoles and bile acids. These metabolites can modulate the absorption and distribution of antibiotics and influence the host immune response to infections [40]. For example, a recent study by Chimerel et al. demonstrated that the gut microbiota-derived metabolite indole propionic acid can enhance the efficacy of certain antibiotics by increasing their penetration into bacterial cells and modulating the host immune response [41].

Use of diagnostic techniques in understanding the role of gut microbiota in antibiotic therapy

The recent advancements in various molecular techniques, such as metagenomics, transcriptomics, and metabolomics, have significantly contributed to our understanding of the role of gut microbiota in antibiotic therapy selection. Metagenomics has enabled the sequencing and analysis of the total genomic content of microbiota, which facilitate the identification of specific bacterial taxa and their functional genes [42]. This approach has led to the discovery of particular genes associated with antibiotic resistance within the gut microbiota, which can guide optimum antibiotic therapy selection to minimize resistance development [43]. Meanwhile, transcriptomics focuses on the analysis of the total RNA transcripts within a microbial community, which reveals the active metabolic pathways and functions of the gut microbiota [44]. Transcriptomic studies have demonstrated the differential expression of genes involved in antibiotic resistance and metabolism in response to antibiotic exposure [45]. This approach can help identify differentially expressed genes involved in antibiotic resistance, metabolism, and host-microbiota interactions. For instance, transcriptomic analyses can reveal the expression of efflux pumps, enzymes, and other factors that contribute to antibiotic resistance in gut bacteria [46]. Metabolomics is a field of study that employs a comprehensive analytical approach to elucidate the small molecules (metabolites) synthesized by the gut microbiota and their host [47]. This discipline has facilitated the identification of specific metabolites, such as short-chain fatty acids that can modulate the absorption and distribution of antibiotics. Furthermore, metabolomic analyses have uncovered the ability of gut microbiota-derived short-chain fatty acids to modulate the function of immune cells and enhance the efficacy of specific antibiotics against bacterial infections [48].

Antibiotic resistance mitigation by gut microbiota

The gut microbiota has been shown to play a significant role in the development and mitigation of antibiotic resistance [49]. Antibiotic resistance occurs when bacteria adapt to the effects of antibiotics, which ultimately render them ineffective. The understanding of the mechanisms by which the gut microbiota mitigates antibiotic resistance is essential in order to develop novel therapeutic strategies to preserve the efficacy of existing antibiotics. The gut microbiota acts as a reservoir for antibiotic resistance genes that can be horizontally transferred between bacterial species. This process allows bacteria to acquire new resistance mechanisms and contribute to the spread of multidrug-resistant pathogens [50].

Competitive Exclusion

One of the mechanisms through which the gut microbiota mitigates antibiotic resistance is competitive exclusion where beneficial commensal bacteria in the gut prevent the colonization and growth of resistant pathogens [51]. Beneficial commensal bacteria in the gut outcompete resistant pathogens by limiting their access to limited resources, such as nutrients. This competitive environment can reduce the opportunity for horizontal gene transfer and limit the spread of resistance genes [52]. By occupying essential niches and utilizing available nutrients, commensal bacteria can outcompete and inhibit the proliferation of antibiotic-resistant bacteria. Furthermore, certain commensal bacteria, such as *Bifidobacterium* and *Lactobacillus* species produce antimicrobial compounds that directly target pathogenic bacteria, which further restricts their growth and minimizes the emergence of resistance [53].

Colonization Resistance

Colonization resistance is a crucial mechanism that aids in reducing antibiotic resistance by impeding the colonization of pathogens in the gut through the production of antimicrobial agents and alteration of the local environment [54]. The commensal microbiota generates bacteriocins, which are protein toxins with antimicrobial properties that selectively inhibit the growth of related bacterial strains, including antibiotic-resistant strains [55]. During colonization resistance, the gut microbiota helps maintain a stable and diverse microbial community, thereby limiting the establishment of antibiotic-resistant pathogens. A diverse gut microbiota also plays a critical role in maintaining a balanced immune response, which can help prevent the overgrowth of resistant bacteria and maintain homeostasis [56]. Commensal bacteria can stimulate the production of host-derived antimicrobial peptides, enhance the function of immune cells, and promote the clearance of pathogens [57]. Antibiotic treatment and other disruptions to the gut microbiota can weaken colonization resistance, which can result in the expansion of resistant pathogens. Therefore, preserving a diverse and healthy gut microbiota is necessary to combat antibiotic resistance [58].

The use of narrow-spectrum antibiotics can preserve colonization resistance and minimize the emergence of antibiotic resistance [59]. Certain commensal bacterial strains have been shown to degrade antibiotics, which reduces their selective pressure on the gut microbiota and limits the development of resistance. This phenomenon can potentially slow down the development of resistance by lowering the exposure of bacteria to antibiotics. However, the extent to which the gut microbiota impacts the pharmacokinetics of antibiotics and the development of resistance remains an area of ongoing investigation [60,61].

Mechanisms of colonization resistance: There are two types of mechanisms in colonization resistance: direct and indirect. The direct mechanism is characterized by the ability of gut microbiota to limit colonization by intestinal pathogens, or the over progression of these pathogens after embedding, and those liberated from the host. Dietary complex carbohydrates and mucins are crucial colonic nutritious means to which commensal types have adapted through definite metabolic paths [62]. Enteropathogens regularly use nourishing sources presented by commensal types [63]. For instance, *Citrobacter rodentium* and *E. coli* may be in rivalry for the absorption of monosaccharides. However, some pathogenic microbes may use gastrointestinal nutrients that commensals cannot digest. For instance, ethanolamine is a nitrogen and carbon source for Enterohaemorrhagic *E. coli* (EHEC), *Salmonella typhimurium*, *Klebsiella*, *C. difficile*,* Listeria monocytogenes* , and *Pseudomonas*, but it cannot be utilized by a maximum of the commensal species [64]. Particularly, EHEC species have advanced metabolic paths for discrete sugar resources, more or less of which are unreachable to commensal *E. coli* [65]. Interestingly, EHEC can be disinterested from its metabolic position, in the existence of two dissimilar strains of *E. coli*, and might fail to form a colony in the gut [66]. 

Cell wall-active bactericidal polypeptides produced by the commensal microbes generally are called bacteriocins. Bacteriocins produced by the Gram-negative bacteria are named microcins, and they have a fine spectrum of action restricted to new Gram-negative microbes [67]. Microcins cause internalization and thus apply their inhibitory influence and restrict the colonization of microbes [68].

In indirect mechanisms, host and commensal flora interactions result in colonization resistance indirectly. This happens through RegIIIγ and angiotensin-4 (antimicrobial peptides), bile acid metabolism, and epithelial barrier mechanism. The production of antimicrobial peptides is regulated by the microbes. Microbiota can stimulate the production of RegIIIγ by stimulating toll-like receptors (TLRs), mainly TLR-4, through the lipopolysaccharides [69]. In the epithelial barrier mechanism, a decrease in the thickness of the mucus layer will result in enhanced pathogen colonization susceptibility. Antibiotic therapy, Western-style diet, and drugs that have impacts on the microbiota will increase colonization susceptibility and thickness of the mucus layer [70].

In the case of bile acid metabolisms, reabsorption of primary bile acids occurs in the terming ileum; however, only a small fraction reaches the large intestine, where the subset of colonic bacteria converts primary bile acids into secondary bile acids. Different bile acids affect differently on the vegetative growth and germination process. For example, taurocholic acid, a primary bile acid, has an impact on germination induction in the *C. difficile* spores, while secondary bile acids are well known to cause an inhibition in the growth of toxin-producing vegetative *C. difficile* [71]. *Clostridium scindens* can convert chenodeoxycholic acid and cholic acid (primary bile acids) to lithocholic acid and deoxycholic acid (secondary bile acids). As a result, *C. scindens* increase resistance to infections by *C. difficile* in both human patients and animals through secondary bile acid-dependent functions [72].

Use of Diagnostic Techniques in Antibiotic Resistance Mitigation by Gut Microbiota

The role of different molecular techniques has been instrumental in studying the role of gut microbiota in mitigating antibiotic resistance. Metagenomic analyses can identify the presence and abundance of antibiotic resistance genes within the gut microbiota [73]. Transcriptomic and proteomic approaches can reveal the expression patterns of resistance genes and help elucidate the mechanisms through which gut microbes interact with antibiotics [74,75]. Metabolomics provides a functional readout of the gut microbiota by identifying metabolites involved in antibiotic resistance or those that could be used to enhance the efficacy of existing antibiotics [76].

Furthermore, recent advances in NGS technologies, such as long-read sequencing and metagenomic assembly, have enabled the identification and characterization of novel antibiotic resistance genes and mobile genetic elements that contribute to the spread of antibiotic resistance within the gut microbiota [77]. These technological advancements have also facilitated the discovery of previously unknown associations between commensal bacteria, pathogens, and host immune responses. In recent times, various research inquiries have employed molecular methodologies to investigate the interplay between the gut microbiota and the management of antibiotic resistance. Metagenomic analyses have been utilized to explore the gut microbiota's capacity to support resistance gene transfer, leading to the revelation that specific environmental factors, such as antibiotic exposure, may foster horizontal gene transfer and the evolution of resistance [78].

Probiotics, Prebiotics, and Fecal Microbiota Transplantation (FMT)

The modulation of gut microbiota is a promising strategy for mitigating antibiotic resistance. Probiotics, prebiotics, and fecal microbiota transplantation (FMT) are potential interventions that can be used for this purpose. Probiotics are living microorganisms that when consumed in appropriate quantities bestow a health benefit on the host by enhancing the resistance of gut microbiota against pathogenic organisms and antibiotic-induced perturbations [79]. Prebiotics, on the other hand, are indigestible food components that selectively promote the growth and activity of beneficial bacteria in the gut [80]. FMT involves the transfer of fecal material containing healthy donor microbiota into a recipient. This technique has been shown to restore the diversity and function of the gut microbiota [81]. Clinical studies have demonstrated the effectiveness of FMT in treating recurrent *C. difficile* infections and reducing the prevalence of antibiotic-resistant bacteria in the gut [82]. A randomized control trial by Huttner et al. showed that a five-day course of non-absorbable antibiotics followed by FMT decreased extended spectrum β-lactamase *Enterobacteriaceae* (ESBL-E) and carbapenemase-producing *Enterobacteriaceae* (CPE) carriage compared with controls [83].

**Figure 2:**
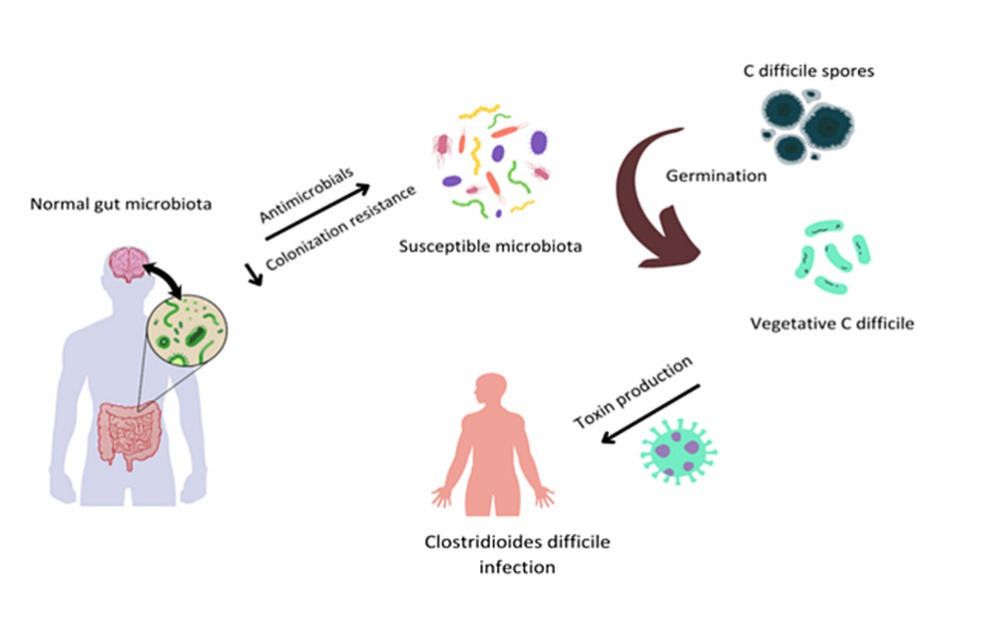
Gut microbiota and Clostridioides difficile infection (CDI)

Additionally, the application of bacteriophages has been proposed as an alternative to conventional antibiotics with the potential to specifically target resistant bacteria without disrupting the overall gut microbial community [84]. Figure 2 shows the impact of *C. difficile* infection on the gut microbiota. 

Clinical applications of gut microbiota research for antibiotic therapy selection

By understanding the complex interactions between the gut microbiota, antibiotics, and host immune responses, clinicians can attain greater acuity in antibiotic selection and implement measures to attenuate resistance. Furthermore, recent advances in omics technologies have facilitated the development of rapid diagnostic tools that can assess the gut microbiota's composition and function, providing real-time information to guide clinical decision-making [85]. Gut microbiota research can contribute to clinical decision-making by pinpointing specific bacterial strains and their susceptibility profiles to antibiotics in an individual's gut microbiota. Such insights can facilitate personalized antibiotic therapy selection, curtail the risk of treatment failure or resistance emergence [86]. For instance, metagenomic sequencing can furnish pertinent information about the prevalence of antibiotic resistance genes in a patient's gut microbial community, thereby empowering clinicians to opt for antibiotics that are less prone to favor resistant bacteria [87]. These tools can help clinicians monitor the effects of antibiotics on the gut microbiota and identify early signs of resistance development or dysbiosis, allowing for the timely intervention and adjustment of antibiotic regimens.

Moreover, research into the use of probiotics, prebiotics, and synbiotics as adjuvant therapies has shown promise in improving antibiotic efficacy and reducing resistance development. In a randomized, double-blind, placebo-controlled trial, Reid et al. demonstrated that the administration of a *Lactobacillus rhamnosus* probiotic alongside antibiotic treatment for bacterial vaginosis resulted in higher cure rates and fewer recurrences compared to the antibiotic treatment alone [88]. Similarly, a study by Timmerman et al. found that synbiotic supplementation in critically ill patients receiving antibiotics led to improved gut permeability, a reduced incidence of antibiotic-associated diarrhea, and a lower abundance of potentially pathogenic bacteria in the gut [89]. The integration of gut microbiota research into clinical decision-making for antibiotic therapy selection and resistance mitigation is still in its early stages, with many challenges remaining. For instance, translating research findings into clinically relevant guidelines and protocols requires a thorough understanding of the complex interactions between the gut microbiota, host immune system, and antibiotics, as well as the development of standardized methodologies for assessing the gut microbiota.

Furthermore, the implementation of gut microbiota-based diagnostics and therapeutics in clinical settings faces several practical barriers, such as the need for specialized equipment, trained personnel, and rigorous quality control measures, to ensure the accuracy and reproducibility of results [90]. Furthermore, the use of FMT and other microbiota-targeted interventions raises questions about the long-term safety and efficacy of these therapies and potential unintended consequences on the recipient's health [91]. Despite the challenges, clinical applications of gut microbiota research for antibiotic therapy selection and resistance mitigation hold great promise for improving patient outcomes. By leveraging cutting-edge molecular techniques and a growing understanding of the complex interactions between the gut microbiota, antibiotics, and host immune responses, clinicians can make more informed decisions about antibiotic selection, dosing, and duration and implement strategies to mitigate resistance and restore gut microbial health.

Current challenges and future directions

The investigation of gut microbiota has the potential to significantly influence the selection of antibiotic therapy and the mitigation of resistance. However, the field faces various challenges. One such challenge is the standardization of techniques and interpretation of results as the field currently lacks universally accepted methodologies and reporting standards. The inconsistency in sample collection, processing, sequencing, and data analysis can result in variations in findings, which obstruct the translation of research outcomes into clinical practice [92]. To address these challenges, researchers are working to develop new analytical methods and establish guidelines for reporting gut microbiota data. Projects, such as the International Human Microbiome Standards (IHMS) initiative's aim to create standardized protocols for sample collection, processing, and data analysis [93]. These protocols enable stronger comparisons across research and facilitate the integration of gut microbiota research into clinical decision-making. Furthermore, the advancement of bioinformatics tools and platforms, such as QIIME 2 and MetaPhlAn, can aid researchers in profiling and analyzing gut microbiota data with precision and facilitate dependable and reproducible outcomes [94,95].

Future research directions in the field include investigating the role of the gut microbiota in antibiotic stewardship and optimizing personalized medicine approaches. Antibiotic stewardship programs could greatly benefit from a better understanding of the role of the gut microbiota in antibiotic response and resistance development [96]. By incorporating gut microbiota research into antibiotic stewardship efforts, healthcare providers can make more informed decisions about when and how to use antibiotics, ultimately preserving their effectiveness and minimizing the risk of resistance [97]. Personalized medicine approaches that utilize individualized gut microbiota profiles to tailor antibiotic therapy have considerable potential for enhancing patient outcomes. The identification of specific bacterial strains and their susceptibility patterns within the gut microbiota of an individual via metagenomic and metatranscriptomic analyses can enable the selection of precisely targeted antibiotic regimens [72]. Furthermore, the exploration of adjuvant therapies, such as probiotics, prebiotics, and synbiotics, may facilitate the development of customized strategies to re-establish gut microbial balance and ameliorate the deleterious effects of antibiotic therapy [98].

## Conclusions

The emerging field of gut microbiota research holds tremendous potential for revolutionizing antibiotic therapy selection and resistance mitigation strategies. The intricate interplay between the gut microbiota, antibiotic therapy, and antibiotic resistance underscores the importance of incorporating this research into clinical practice. By addressing the challenges and future directions associated with this field, including standardization of methodologies, ethical considerations, and interdisciplinary collaboration, we can pave the way for the integration of gut microbiota research into antibiotic therapy and resistance mitigation efforts. Harnessing the power of gut microbiota research can not only optimize antibiotic use and preserve the efficacy of these lifesaving drugs but also significantly contribute to combating the global threat of antibiotic resistance. Furthermore, the development and validation of novel diagnostic tools, therapeutic interventions, and predictive models will facilitate the practical implementation of gut microbiota research findings in clinical settings. By maintaining an agile and responsive research environment, the scientific community can swiftly adapt to new findings and challenges, driving the field of gut microbiota research forward and making significant strides toward better antibiotic therapy selection and resistance mitigation.
